# Tris(ethanol-κ*O*)tris­(picrato-κ^2^ *O* ^1^,*O* ^2^)lanthanum(III) tri-2-pyridylamine solvate

**DOI:** 10.1107/S1600536808019296

**Published:** 2008-07-05

**Authors:** Eric J. Chan

**Affiliations:** a Research School of Chemistry, Building 35, Australian National University, Canberra, ACT 0200, Australia

## Abstract

The title compound, [La(C_6_H_2_N_3_O_7_)_3_(C_2_H_6_O)_3_]·C_15_H_12_N_4_, has two mol­ecular building blocks, namely the neutral mononuclear adduct of lanthanum picrate with ethanol [*i.e.* La(pic)_3_:EtOH (1:3); La(pic)_3_ = lanthanum picrate and EtOH = ethanol] and the oligodentate aromatic nitro­gen base tri-2-pyridylamine (tpa). The asymmetric unit contains two formula units. The compound was prepared during an investigation of the stereochemistry of lanthanoid picrate complexes with *O*-donor ligands. The metal–ligand adduct adopts a nine-coordinate tricapped trigonal-prismatic metal atom environment. The stereochemical arrangement of the ligands about the metal core is typical of a *fac*-isomer with stoichiometry *M*(bidentate)_3_(monodentate)_3_. Face-to-face hydrogen bonds are found between the tpa mol­ecule and the ethanol ligands. One ethanol ligand is disordered over two positions, with site occupancy factors of *ca* 0.7 and 0.3. The oxygen atoms of a nitro group are also disordered over two positions; the site occupancy factors are *ca* 0.6 and 0.4.

## Related literature

The compounds Ln(NO_3_)_3_(EtOH)_3_·tpa display identical structural features and are produced by a similar method of synthesis (Nagao *et al.*, 2004 ▶). For the stereochemistry of compounds with stoichiometry Ln(pic)_3_(unidentate)_3_ (pic = picrate), see: Chan (2006 ▶). For an inter­pretation of the inter­molecular inter­actions between metal complexes with picrate ligands, see: Harrowfield (1996 ▶). For the preparation of lanthanoid picrate hydrates, see: Harrowfield *et al.* (1994 ▶). For the preferred stereochemical arrangement of multidentate ligands encompassing a nine-coordinate metal atom environment, see: Kepert (1986 ▶).
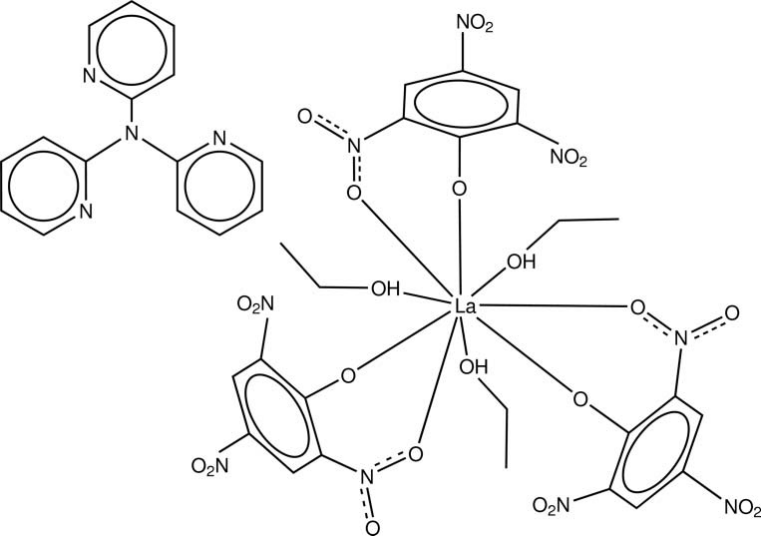


## Experimental

### 

#### Crystal data

[La(C_6_H_2_N_3_O_7_)_3_(C_2_H_6_O)_3_]·C_15_H_12_N_4_
*M* *_r_* = 1209.69
Triclinic, 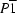

*a* = 15.7554 (19) Å
*b* = 16.4752 (14) Å
*c* = 20.427 (4) Å
α = 101.714 (11)°
β = 111.610 (14)°
γ = 90.676 (8)°
*V* = 4805.1 (13) Å^3^
*Z* = 4
Mo *K*α radiation
μ = 0.99 mm^−1^
*T* = 100 (2) K
0.50 × 0.18 × 0.03 mm

#### Data collection

Oxford Diffraction Xcalibur diffractometer
Absorption correction: multi-scan (*CrysAlis RED*; Oxford Diffraction, 2006 ▶) *T*_min_ = 0.773, *T*_max_ = 0.970
58832 measured reflections
28469 independent reflections
14209 reflections with *I* > 2σ(*I*)
*R*_int_ = 0.049

#### Refinement

*R*[*F* ^2^ > 2σ(*F* ^2^)] = 0.043
*wR*(*F* ^2^) = 0.086
*S* = 0.84
28469 reflections
1419 parameters
104 restraints
H-atom parameters constrained
Δρ_max_ = 2.85 e Å^−3^
Δρ_min_ = −0.75 e Å^−3^

### 

Data collection: *CrysAlis CCD* (Oxford Diffraction, 2006 ▶); cell refinement: *CrysAlis RED* (Oxford Diffraction, 2006 ▶); data reduction: *CrysAlis RED*; program(s) used to solve structure: *SIR92* (Altomare *et al.*, 1994 ▶); program(s) used to refine structure: *SHELXL97* (Sheldrick, 2008 ▶); molecular graphics: *Xtal3.7* (Hall *et al.*, 2001 ▶); software used to prepare material for publication: *Xtal3.7*.

## Supplementary Material

Crystal structure: contains datablocks I, global. DOI: 10.1107/S1600536808019296/zl2121sup1.cif

Structure factors: contains datablocks I. DOI: 10.1107/S1600536808019296/zl2121Isup2.hkl

Additional supplementary materials:  crystallographic information; 3D view; checkCIF report

**Fig. 1.**
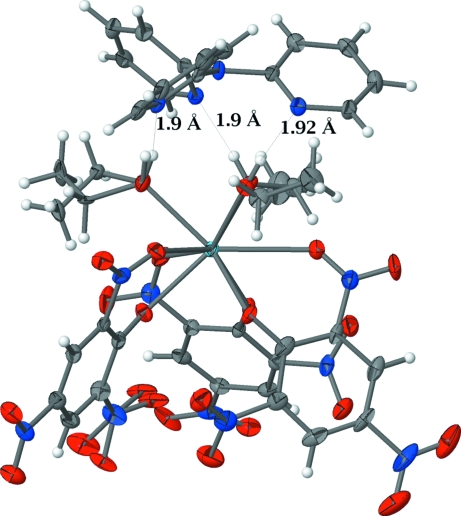
View of the molecular structure of (I) depicting the tris(2-pyridyl)amine molecule interacting with the (C6H2N3O7-O,O')3(C2H6O-O)3La(III) adduct through O—H···N hydrogen bonds. 50% probability displacement ellipsoids are shown for non-H atoms. Enhanced figure is accessible at http://submission.iucr.org/jtkt/serve/z/u4xwab7OUyDKQ3um/zz0000/0/

Enhanced figure: interactive version of Fig. 1

## Figures and Tables

**Table 1:** Hydrogen-bond geometry (Å, °)

*D*—H⋯*A*	*D*—H	H⋯*A*	*D*⋯*A*	*D*—H⋯*A*
O011—H011⋯N111	0.84	1.90	2.740 (4)	175
O021—H021⋯N121	0.84	1.92	2.747 (3)	170
O031—H031⋯N131	0.84	1.90	2.693 (3)	158
O041—H041⋯N241^i^	0.84	1.88	2.721 (4)	179
O051—H051⋯N251^i^	0.84	1.93	2.769 (3)	172
O061—H061⋯N261^i^	0.84	1.85	2.692 (4)	175

Symmetry code: (i) 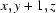.

**Fig. 2.**
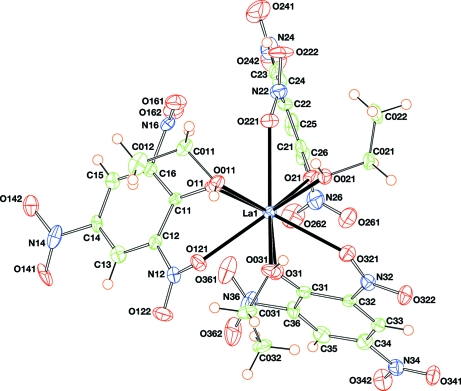
Labelled ORTEP diagram of the tris(ethanol-O)tris(picrato-O,O')lanthanum(III) neutral mononuclear adduct projected through the triangular faces of its 9-coordinate tri-capped trigonal prismatic metal environment. 50% probability displacement ellipsoids are shown for non-H atoms.

**Fig. 3.**
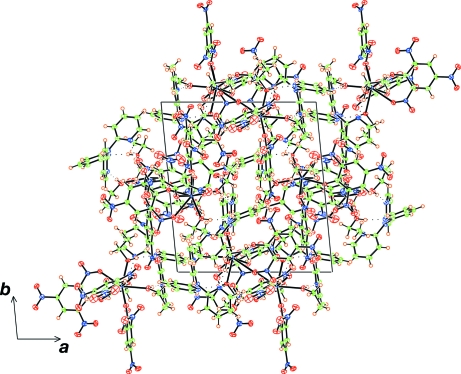
Packing diagram of (I) viewed down the c-axis. The O—H···N hydrogen bonds are shown as dotted lines. 50% probability displacement ellipsoids are shown for non-H atoms.

## supplementary crystallographic information

### Comment

The title compound crystallizes in space group *P*1 with two independent groups of (C_6_H_2_N_3_O_7_-*O*,*O'*)_3_La(*O*-C_2_H_6_O)_3_. (C_5_H_4_N)_3_N, (I), in the asymmetric unit (*Z*=4). Each individual bimolecular cluster comprises the metal-ligand adduct La(pic)_3_(EtOH)_3_ hydrogen bonded to the aromatic nitrogen base molecule tpa through a "face to face" arrangement in which the pyridine nitrogen atoms of the tpa molecules act as hydrogen bond acceptors and the hydroxy groups of ethanol ligands act as hydrogen bond donors (see Fig. 1 and Fig. 3). The primary coordination sphere of lanthanum is nine-coordinate, consisting only of ligand oxygen donor atoms, adopting the *"fac"* isomeric form of a tri-capped trigonal prism in which all three unidentate ethanol ligands occupy mutually *"cis"*-sites of one triangular face and the bidentate *O,O'*–picrate anions chelating through the phenoxy oxygen atoms at sites of the opposite triangular face and with an adjacent *O*–nitro oxygen atom at the capping site of each rectangular face (see Fig. 2).

The bimolecular cluster has a pseudo-threefold axis normal to the triangular faces of the tri-capped trigonal prism disposed about the lanthanum core. There is an obtuse (greater than 120°) nitro–*O*—La—*O*–nitro angle (approx. 125°) when compared with other angles associated with picrate nitro group oxygen atoms at the capping sites of the tri-capped trigonal prism (see Fig. 2, values for O121—La1—O321, O221 being 116.62 (7), 125.17 (7)° respectively with O321—La1—O221 being 117.88 (7)° and values for O421—La2—O621, O521 being 115.75 (7), 126.73 (7)° respectively with O621—La2—O521 being 117.28 (7)°). Presumably, the length of the corresponding rectangular edge (phenoxide oxygen and ethanol oxygen donor atoms) of the trigonal prism opposite to this angle becomes the shortest rectangular edge distance as a result of minimizing strain (contact distances for O11···O011, O21···O021 and O31···O031 being 3.217 (3), 3.341 (3) and 3.400 (4) Å respectively, with contact distances for O41···O041, O51···O051 and O61···O061 being 3.115 (4), 3.397 (4) and 3.483 (4) Å respectively). This observation exists concomitantly with a distortion of the inter-planar dihedral angle between the tpa pyridyl group, whose nitrogen atom is hydrogen bonded to the ethanol ligand which is associated with the above mentioned shorter rectangular edge, and the central NC_3_ plane of the tpa molecule. Noticeably, the dihedral angles between the NC_3_ and C_5_N planes of tpa which comprise nitrogen atoms labelled N111, N121 and N131 are 44.7 (1), 39.0 (1) and 40.0 (1)° respectively. The corresponding dihedral angles found in the second set of molecular coordinates for planes comprising nitrogen atoms N241, N251 and N261 are 45.7 (1), 40.6 (1) and 37.4 (1)° respectively. In agreement with the previous statement the angles for N111 and N241 are seen to be significantly larger.

### Experimental

Using a 1:1 mole ratio, hydrated La(pic)_3_ (Harrowfield *et al.*,, 1994) and tpa was dissolved in a suitable volume of 70% *v*/*v* ethanol in triethyl orthoformate (used in the synthesis as a dehydrating agent). The mixture was then heated under reflux for 1 h ensuring formation of complex is complete. The solution was then filtered while hot into a Shlenk tube fitted with vacuum and nitrogen outlet. The solvent was removed under vacuum until the contents were sufficiently concentrated, the product was then allowed to cool slowly until yellow crystals deposited.

### Refinement

All H atoms, with the exception of those associated with the hydroxy groups of the ethanol molecules, were positioned geometrically and refined using a riding model with C—H = 0.95–0.99 Å, and with *U*_iso_(H) = 1.2 (1.5 for methyl groups) times *U*_eq_(C). The hydroxy H atoms of the ethanol molecules were, in the early stages of refinement, built geometrically as idealized OH groups and refined using a riding model (allowing rotation about the C—O bond) until the mean shift/e.s.d. was at a minimum of 0.01, with the C—O—H angle tetrahedral and O—H = 0.80–0.85 Å, and with *U*_iso_(H) = 1.5 times *U*_eq_(O). A further five final cycles of refinement were performed with the hydroxy H atom positions fixed in order to allow hydroxy oxygen atom to lanthanum metal atom bond lengths with associated angles to be included in the connectivity list. Quasi-in-plane orientational disorder was exhibited for the totality of ethanol ligand "03" (with the exception of its La-bound oxygen atom "O031"). All 1,2– and 1,3– distances for the disordered atoms were restrained so that both fragments would have similar geometries (*i.e.* the SAME restraint, using the default 0.02 s.u. values). The sum of the site occupation parameters for both disordered groups was constrained to unity during refinement giving a major site occupation component value of 0.664 (8) with that of the minor component value complementary. All atoms of the disordered ethanol group closer than 1.7 Å were restrained to have similar U_ij_ components (i.e. the SIMU restraint was applied) with the default 0.04 (central C atom) and 0.08 (terminal C and O atoms) s.u. values. The oxygen atoms of the "36" picrate nitro group exhibit rotational disorder around the N—C bond. During refinement the N—O distances were restrained to be the same (*i.e.* the SADI restraint, using the default 0.02 s.u. value) and atoms of each of the CNO_2_ fragments (atoms C36 and N36 both being a part of each planar group) were restrained to lie in a common plane (*i.e.* the FLAT restraint, using the default 0.1 s.u. value). The sum of site occupancies was constrained to unity, oxygen fragments refining to a major site occupation value of 0.58 (2) with a minor component value complementary. All atoms of the disordered nitro group closer than 1.7 Å were restrained to have similar U_ij_ components (i.e. the SIMU restraint was applied) with the default 0.04 (central N atom) and 0.08 (terminal O atoms) s.u. values.

### Crystal data

**Table:**

[La(C_6_H_2_N_3_O_7_)_3_(C_2_H_6_O)_3_]·C_15_H_12_N_4_	*Z* = 4
*M**_r_* = 1209.69	*F*_000_ = 2440
Triclinic, *P*1	*D*_x_ = 1.672 Mg m^−^^3^
Hall symbol: -P 1	Mo *K*α radiation λ = 0.71073 Å
*a* = 15.7554 (19) Å	Cell parameters from 16526 reflections
*b* = 16.4752 (14) Å	θ = 2.6–31.2º
*c* = 20.427 (4) Å	µ = 0.99 mm^−^^1^
α = 101.714 (11)º	*T* = 100 (2) K
β = 111.610 (14)º	Plate, yellow
γ = 90.676 (8)º	0.50 × 0.18 × 0.03 mm
*V* = 4805.1 (13) Å^3^	

### Data collection

**Table:**

Oxford Diffraction Xcalibur diffractometer	28469 independent reflections
Radiation source: fine-focus sealed tube	14209 reflections with *I* > 2σ(*I*)
Monochromator: graphite	*R*_int_ = 0.049
Detector resolution: 16.0009 pixels mm^-1^	θ_max_ = 31.2º
*T* = 100(2) K	θ_min_ = 2.6º
ω scans	*h* = −22→22
Absorption correction: multi-scan(CrysAlis RED; Oxford Diffraction, 2006)	*k* = −23→23
*T*_min_ = 0.773, *T*_max_ = 0.970	*l* = −29→29
58832 measured reflections	

### Refinement

**Table:**

Refinement on *F*^2^	Secondary atom site location: difference Fourier map
Least-squares matrix: full	Hydrogen site location: inferred from neighbouring sites
*R*[*F*^2^ > 2σ(*F*^2^)] = 0.043	H-atom parameters constrained
*wR*(*F*^2^) = 0.086	*w* = 1/[σ^2^(*F*_o_^2^) + (0.031*P*)^2^] where *P* = (*F*_o_^2^ + 2*F*_c_^2^)/3
*S* = 0.84	(Δ/σ)_max_ = 0.047
28469 reflections	Δρ_max_ = 2.85 e Å^−^^3^
1419 parameters	Δρ_min_ = −0.75 e Å^−^^3^
104 restraints	Extinction correction: none
Primary atom site location: structure-invariant direct methods	

### Special details

**Table:**

Experimental. CrysAlis RED (Oxford Diffraction, 2006) Empirical absorption correction using spherical harmonics as implemented in the SCALE3 ABSPACK scaling algorithm.
Geometry. All e.s.d.'s (except the e.s.d. in the dihedral angle between two l.s. planes) are estimated using the full covariance matrix. The cell e.s.d.'s are taken into account individually in the estimation of e.s.d.'s in distances, angles and torsion angles; correlations between e.s.d.'s in cell parameters are only used when they are defined by crystal symmetry. An approximate (isotropic) treatment of cell e.s.d.'s is used for estimating e.s.d.'s involving l.s. planes.
Refinement. Refinement of *F*^2^ against ALL reflections. The weighted *R*-factor *wR* and goodness of fit *S* are based on *F*^2^, conventional *R*-factors *R* are based on *F*, with *F* set to zero for negative *F*^2^. The threshold expression of *F*^2^ > σ(*F*^2^) is used only for calculating *R*-factors(gt) *etc*. and is not relevant to the choice of reflections for refinement. *R*-factors based on *F*^2^ are statistically about twice as large as those based on *F*, and *R*- factors based on ALL data will be even larger.

### Fractional atomic coordinates and isotropic or equivalent isotropic displacement parameters (Å^2^)

**Table:**

	*x*	*y*	*z*	*U*_iso_*/*U*_eq_	Occ. (<1)
La1	0.860784 (13)	0.552421 (12)	0.752304 (10)	0.01733 (5)	
O11	0.84562 (14)	0.40793 (13)	0.69097 (11)	0.0192 (5)	
C11	0.8443 (2)	0.33456 (19)	0.70167 (17)	0.0162 (7)	
C12	0.8586 (2)	0.31263 (19)	0.76897 (18)	0.0192 (7)	
C13	0.8654 (2)	0.2323 (2)	0.7780 (2)	0.0231 (8)	
H13	0.8804	0.2213	0.8247	0.028*	
C14	0.8503 (2)	0.16812 (19)	0.71953 (19)	0.0203 (7)	
C15	0.8328 (2)	0.1827 (2)	0.65141 (19)	0.0211 (8)	
H15	0.8232	0.1379	0.6112	0.025*	
C16	0.8296 (2)	0.2627 (2)	0.64365 (18)	0.0193 (7)	
N12	0.86660 (19)	0.37655 (18)	0.83211 (16)	0.0244 (7)	
O121	0.83658 (16)	0.44447 (14)	0.82365 (12)	0.0256 (5)	
O122	0.89884 (19)	0.36014 (15)	0.89202 (13)	0.0386 (7)	
N14	0.84826 (19)	0.08238 (18)	0.7297 (2)	0.0295 (8)	
O141	0.87984 (16)	0.06817 (16)	0.78795 (16)	0.0373 (7)	
O142	0.81122 (16)	0.02741 (14)	0.67350 (14)	0.0303 (6)	
N16	0.8119 (2)	0.27897 (17)	0.57155 (15)	0.0238 (7)	
O161	0.75047 (17)	0.32233 (15)	0.54706 (13)	0.0333 (6)	
O162	0.86114 (17)	0.24768 (15)	0.54005 (13)	0.0340 (6)	
O21	0.95890 (14)	0.55337 (14)	0.68478 (12)	0.0227 (5)	
C21	0.9606 (2)	0.5047 (2)	0.62887 (19)	0.0234 (8)	
C22	0.8859 (2)	0.4847 (2)	0.56022 (18)	0.0221 (8)	
C23	0.8899 (3)	0.4340 (2)	0.49848 (19)	0.0286 (9)	
H23	0.8392	0.4252	0.4536	0.034*	
C24	0.9694 (3)	0.3969 (2)	0.5041 (2)	0.0294 (9)	
C25	1.0435 (3)	0.4080 (2)	0.5692 (2)	0.0317 (9)	
H25	1.0963	0.3793	0.5728	0.038*	
C26	1.0389 (2)	0.4614 (2)	0.6284 (2)	0.0246 (8)	
N22	0.7984 (2)	0.51949 (17)	0.55199 (17)	0.0259 (7)	
O221	0.77665 (15)	0.53675 (14)	0.60512 (12)	0.0256 (6)	
O222	0.74856 (18)	0.52775 (17)	0.49225 (14)	0.0414 (7)	
N24	0.9739 (3)	0.3425 (2)	0.4392 (2)	0.0439 (10)	
O241	0.9067 (2)	0.33343 (19)	0.38276 (18)	0.0616 (10)	
O242	1.0466 (2)	0.31122 (18)	0.44525 (17)	0.0598 (9)	
N26	1.1194 (2)	0.4714 (2)	0.69646 (18)	0.0330 (8)	
O261	1.14614 (16)	0.54218 (16)	0.73386 (14)	0.0362 (7)	
O262	1.15419 (18)	0.40807 (18)	0.71157 (16)	0.0479 (8)	
O31	1.00390 (15)	0.51721 (13)	0.83415 (12)	0.0236 (5)	
C31	1.0896 (2)	0.5396 (2)	0.86261 (18)	0.0206 (8)	
C32	1.1283 (2)	0.62516 (19)	0.88504 (17)	0.0170 (7)	
C33	1.2209 (2)	0.6484 (2)	0.92038 (17)	0.0213 (8)	
H33	1.2434	0.7056	0.9348	0.026*	
C34	1.2801 (2)	0.5889 (2)	0.93453 (19)	0.0242 (8)	
C35	1.2499 (2)	0.5048 (2)	0.91411 (19)	0.0261 (8)	
H35	1.2922	0.4641	0.9247	0.031*	
C36	1.1579 (2)	0.48161 (19)	0.87832 (19)	0.0248 (8)	
N32	1.0704 (2)	0.69303 (17)	0.86917 (15)	0.0219 (6)	
O321	0.98921 (15)	0.67753 (13)	0.82651 (13)	0.0247 (5)	
O322	1.10475 (15)	0.76525 (14)	0.89742 (13)	0.0278 (6)	
N34	1.37835 (19)	0.61448 (19)	0.97202 (16)	0.0254 (7)	
O341	1.40413 (15)	0.68896 (15)	0.99330 (13)	0.0298 (6)	
O342	1.43025 (16)	0.55841 (15)	0.98042 (14)	0.0350 (6)	
N36	1.1311 (2)	0.39154 (18)	0.85566 (18)	0.0359 (8)	
O361	1.0590 (7)	0.3646 (14)	0.8045 (8)	0.039 (3)	0.59 (2)
O362	1.1776 (6)	0.3455 (4)	0.8937 (7)	0.048 (3)	0.59 (2)
O363	1.0508 (9)	0.366 (2)	0.8229 (11)	0.039 (3)	0.41 (2)
O364	1.1983 (6)	0.3466 (5)	0.8618 (9)	0.034 (4)	0.41 (2)
O011	0.69116 (14)	0.52258 (13)	0.71509 (12)	0.0237 (5)	
H011	0.6645	0.5450	0.7419	0.036*	
C011	0.6261 (2)	0.4638 (2)	0.65326 (19)	0.0256 (8)	
H01A	0.6566	0.4406	0.6199	0.031*	
H01B	0.5745	0.4934	0.6270	0.031*	
C012	0.5896 (3)	0.3944 (3)	0.6747 (2)	0.0548 (13)	
H01C	0.5463	0.3563	0.6315	0.082*	
H01D	0.5580	0.4169	0.7068	0.082*	
H01E	0.6403	0.3643	0.7000	0.082*	
O021	0.81139 (14)	0.68541 (13)	0.71535 (12)	0.0208 (5)	
H021	0.7554	0.6928	0.7001	0.031*	
C021	0.8602 (2)	0.7417 (2)	0.69256 (19)	0.0235 (8)	
H02A	0.9150	0.7164	0.6879	0.028*	
H02B	0.8814	0.7942	0.7299	0.028*	
C022	0.8015 (2)	0.7608 (2)	0.6218 (2)	0.0332 (9)	
H02C	0.8372	0.7986	0.6081	0.050*	
H02D	0.7481	0.7872	0.6265	0.050*	
H02E	0.7811	0.7090	0.5845	0.050*	
O031	0.83140 (16)	0.62240 (13)	0.86196 (12)	0.0261 (6)	0.661 (8)
H031	0.8010	0.6632	0.8534	0.039*	0.661 (8)
C031	0.8293 (4)	0.6018 (3)	0.9284 (3)	0.0241 (15)	0.661 (8)
H03A	0.7930	0.5477	0.9169	0.029*	0.661 (8)
H03B	0.8015	0.6453	0.9529	0.029*	0.661 (8)
C032	0.9264 (4)	0.5973 (4)	0.9753 (3)	0.0297 (17)	0.661 (8)
H03C	0.9288	0.5841	1.0206	0.044*	0.661 (8)
H03D	0.9614	0.6511	0.9858	0.044*	0.661 (8)
H03E	0.9528	0.5539	0.9504	0.044*	0.661 (8)
O033	0.83140 (16)	0.62240 (13)	0.86196 (12)	0.0261 (6)	0.339 (8)
H033	0.8123	0.6698	0.8628	0.039*	0.339 (8)
C033	0.9030 (7)	0.6198 (7)	0.9377 (5)	0.023 (3)	0.339 (8)
H03F	0.9475	0.5793	0.9331	0.028*	0.339 (8)
H03G	0.9369	0.6753	0.9623	0.028*	0.339 (8)
C034	0.8519 (7)	0.5942 (8)	0.9799 (6)	0.039 (4)	0.339 (8)
H03H	0.8952	0.5926	1.0284	0.058*	0.339 (8)
H03I	0.8193	0.5389	0.9554	0.058*	0.339 (8)
H03J	0.8077	0.6344	0.9837	0.058*	0.339 (8)
N100	0.63018 (17)	0.71629 (16)	0.77801 (14)	0.0186 (6)	
N111	0.60883 (17)	0.59039 (17)	0.80784 (15)	0.0206 (6)	
C112	0.5936 (2)	0.5476 (2)	0.85276 (19)	0.0249 (8)	
H112	0.5883	0.4884	0.8397	0.030*	
C113	0.5853 (2)	0.5851 (2)	0.91656 (19)	0.0240 (8)	
H113	0.5734	0.5526	0.9462	0.029*	
C114	0.5945 (2)	0.6707 (2)	0.93635 (19)	0.0256 (8)	
H114	0.5903	0.6982	0.9805	0.031*	
C115	0.6101 (2)	0.7161 (2)	0.89125 (18)	0.0244 (8)	
H115	0.6160	0.7753	0.9034	0.029*	
C116	0.6169 (2)	0.6733 (2)	0.82803 (17)	0.0178 (7)	
N121	0.62439 (18)	0.68932 (16)	0.65925 (15)	0.0220 (6)	
C122	0.5771 (2)	0.6647 (2)	0.58794 (19)	0.0248 (8)	
H122	0.6083	0.6678	0.5565	0.030*	
C123	0.4864 (2)	0.6354 (2)	0.5580 (2)	0.0296 (9)	
H123	0.4560	0.6169	0.5071	0.035*	
C124	0.4395 (2)	0.6333 (2)	0.6038 (2)	0.0277 (8)	
H124	0.3765	0.6132	0.5847	0.033*	
C125	0.4861 (2)	0.6609 (2)	0.67723 (19)	0.0227 (8)	
H125	0.4556	0.6610	0.7096	0.027*	
C126	0.5789 (2)	0.68842 (19)	0.70310 (17)	0.0184 (7)	
N131	0.77762 (18)	0.77605 (16)	0.85513 (14)	0.0206 (6)	
C132	0.8455 (2)	0.8387 (2)	0.87808 (19)	0.0254 (8)	
H132	0.9014	0.8348	0.9162	0.030*	
C133	0.8375 (2)	0.9074 (2)	0.84895 (18)	0.0241 (8)	
H133	0.8874	0.9491	0.8651	0.029*	
C134	0.7552 (2)	0.9146 (2)	0.79552 (19)	0.0259 (8)	
H134	0.7477	0.9620	0.7748	0.031*	
C135	0.6838 (2)	0.8532 (2)	0.77220 (18)	0.0226 (8)	
H135	0.6263	0.8575	0.7360	0.027*	
C136	0.6987 (2)	0.78497 (19)	0.80338 (17)	0.0178 (7)	
La2	0.633752 (13)	0.916258 (12)	0.243353 (10)	0.01671 (5)	
O41	0.64846 (14)	0.80202 (14)	0.30240 (11)	0.0198 (5)	
C41	0.6527 (2)	0.7237 (2)	0.29249 (18)	0.0219 (8)	
C42	0.6402 (2)	0.66825 (19)	0.22517 (17)	0.0172 (7)	
C43	0.6363 (2)	0.5827 (2)	0.21597 (19)	0.0207 (7)	
H43	0.6237	0.5480	0.1696	0.025*	
C44	0.6511 (2)	0.5485 (2)	0.27501 (19)	0.0207 (7)	
C45	0.6658 (2)	0.5979 (2)	0.34318 (18)	0.0219 (8)	
H45	0.6743	0.5736	0.3832	0.026*	
C46	0.6676 (2)	0.6820 (2)	0.35048 (17)	0.0175 (7)	
N42	0.63182 (18)	0.69970 (17)	0.16134 (15)	0.0206 (6)	
O421	0.65665 (15)	0.77426 (13)	0.16916 (12)	0.0226 (5)	
O422	0.60455 (17)	0.65152 (15)	0.10237 (13)	0.0335 (6)	
N44	0.65393 (18)	0.45574 (17)	0.26500 (17)	0.0196 (6)	
O441	0.62705 (16)	0.41551 (15)	0.20558 (16)	0.0340 (7)	
O442	0.68841 (16)	0.43226 (14)	0.32138 (14)	0.0306 (6)	
N46	0.6819 (2)	0.73521 (18)	0.42171 (16)	0.0262 (7)	
O461	0.74305 (17)	0.79230 (15)	0.44626 (13)	0.0338 (6)	
O462	0.63206 (17)	0.71809 (16)	0.45197 (13)	0.0366 (6)	
O51	0.53601 (14)	0.94981 (13)	0.31108 (12)	0.0215 (5)	
C51	0.5352 (2)	0.93254 (19)	0.36869 (18)	0.0184 (7)	
C52	0.6101 (2)	0.9501 (2)	0.43716 (18)	0.0219 (8)	
C53	0.6056 (2)	0.9327 (2)	0.49947 (18)	0.0249 (8)	
H53	0.6565	0.9479	0.5441	0.030*	
C54	0.5259 (3)	0.8932 (2)	0.49544 (19)	0.0263 (8)	
C55	0.4513 (2)	0.8701 (2)	0.4309 (2)	0.0265 (8)	
H55	0.3977	0.8406	0.4285	0.032*	
C56	0.4557 (2)	0.8906 (2)	0.37010 (19)	0.0229 (8)	
N52	0.6967 (2)	0.98864 (17)	0.44359 (17)	0.0251 (7)	
O521	0.71871 (14)	0.97801 (14)	0.39039 (12)	0.0234 (5)	
O522	0.74768 (17)	1.02960 (15)	0.50342 (14)	0.0354 (6)	
N54	0.5208 (3)	0.8752 (2)	0.56113 (19)	0.0368 (8)	
O541	0.5884 (2)	0.89389 (19)	0.61692 (15)	0.0522 (8)	
O542	0.4460 (2)	0.84492 (18)	0.55689 (15)	0.0500 (8)	
N56	0.37638 (19)	0.8642 (2)	0.30236 (16)	0.0283 (7)	
O561	0.34683 (16)	0.91519 (16)	0.26496 (13)	0.0335 (6)	
O562	0.34174 (17)	0.79208 (17)	0.28666 (15)	0.0445 (7)	
O61	0.49117 (14)	0.83816 (13)	0.15971 (12)	0.0204 (5)	
C61	0.4058 (2)	0.8434 (2)	0.13435 (17)	0.0180 (7)	
C62	0.3641 (2)	0.9168 (2)	0.11297 (18)	0.0207 (8)	
C63	0.2705 (2)	0.9208 (2)	0.08079 (17)	0.0196 (7)	
H63	0.2467	0.9701	0.0669	0.023*	
C64	0.2121 (2)	0.8507 (2)	0.06938 (16)	0.0174 (7)	
C65	0.2457 (2)	0.7779 (2)	0.08790 (17)	0.0191 (7)	
H65	0.2050	0.7303	0.0789	0.023*	
C66	0.3380 (2)	0.7753 (2)	0.11914 (18)	0.0212 (7)	
N62	0.42103 (18)	0.99304 (16)	0.12567 (15)	0.0194 (6)	
O621	0.50192 (15)	1.00287 (13)	0.16861 (12)	0.0230 (5)	
O622	0.38583 (15)	1.04725 (14)	0.09305 (13)	0.0316 (6)	
N64	0.11342 (18)	0.85389 (18)	0.03600 (15)	0.0227 (7)	
O641	0.08434 (15)	0.91680 (14)	0.01608 (13)	0.0265 (6)	
O642	0.06349 (15)	0.79255 (15)	0.03100 (13)	0.0315 (6)	
N66	0.3699 (2)	0.69686 (18)	0.13932 (17)	0.0275 (7)	
O661	0.44218 (16)	0.69852 (15)	0.19101 (15)	0.0351 (6)	
O662	0.32053 (17)	0.63251 (15)	0.10357 (15)	0.0397 (7)	
O041	0.80292 (14)	0.90418 (13)	0.28443 (11)	0.0210 (5)	
H041	0.8297	0.9108	0.2569	0.031*	
C041	0.8663 (2)	0.8781 (2)	0.34645 (19)	0.0250 (8)	
H04A	0.8338	0.8681	0.3777	0.030*	
H04B	0.9160	0.9234	0.3748	0.030*	
C042	0.9081 (3)	0.7998 (2)	0.3252 (2)	0.0490 (12)	
H04C	0.9498	0.7840	0.3688	0.073*	
H04D	0.9422	0.8101	0.2957	0.073*	
H04E	0.8592	0.7547	0.2974	0.073*	
O051	0.68822 (14)	1.06876 (12)	0.27962 (12)	0.0190 (5)	
H051	0.7450	1.0827	0.2983	0.028*	
C051	0.6404 (2)	1.13673 (19)	0.30115 (18)	0.0215 (8)	
H05A	0.5846	1.1141	0.3053	0.026*	
H05B	0.6210	1.1697	0.2635	0.026*	
C052	0.6994 (2)	1.1925 (2)	0.37188 (19)	0.0288 (9)	
H05C	0.6648	1.2378	0.3848	0.043*	
H05D	0.7542	1.2158	0.3676	0.043*	
H05E	0.7178	1.1603	0.4095	0.043*	
O061	0.67246 (14)	0.93827 (13)	0.13884 (12)	0.0204 (5)	
H061	0.6920	0.9875	0.1431	0.031*	
C061	0.6547 (2)	0.8914 (2)	0.06678 (17)	0.0217 (8)	
H06A	0.6791	0.8365	0.0689	0.026*	
H06B	0.6864	0.9214	0.0436	0.026*	
C062	0.5527 (2)	0.8792 (2)	0.02221 (19)	0.0268 (8)	
H06C	0.5417	0.8463	−0.0264	0.040*	
H06D	0.5290	0.9335	0.0189	0.040*	
H06E	0.5214	0.8497	0.0453	0.040*	
N200	0.87961 (17)	0.07030 (16)	0.22678 (14)	0.0190 (6)	
N241	0.88834 (17)	−0.07251 (16)	0.19476 (15)	0.0186 (6)	
C242	0.8960 (2)	−0.1403 (2)	0.14801 (19)	0.0231 (8)	
H242	0.8947	−0.1932	0.1596	0.028*	
C243	0.9055 (2)	−0.1362 (2)	0.08426 (19)	0.0244 (8)	
H243	0.9108	−0.1849	0.0525	0.029*	
C244	0.9072 (2)	−0.0592 (2)	0.06807 (18)	0.0230 (8)	
H244	0.9137	−0.0544	0.0245	0.028*	
C245	0.8995 (2)	0.0114 (2)	0.11468 (17)	0.0199 (7)	
H245	0.9007	0.0648	0.1042	0.024*	
C246	0.8900 (2)	0.0010 (2)	0.17702 (18)	0.0194 (7)	
N251	0.87729 (18)	0.09980 (16)	0.34369 (15)	0.0215 (6)	
C252	0.9208 (2)	0.1091 (2)	0.41534 (18)	0.0233 (8)	
H252	0.8872	0.1256	0.4453	0.028*	
C253	1.0108 (2)	0.0960 (2)	0.44737 (19)	0.0292 (9)	
H253	1.0391	0.1036	0.4983	0.035*	
C254	1.0599 (2)	0.0713 (2)	0.40365 (19)	0.0280 (8)	
H254	1.1220	0.0599	0.4243	0.034*	
C255	1.0178 (2)	0.0635 (2)	0.33003 (18)	0.0233 (8)	
H255	1.0504	0.0486	0.2991	0.028*	
C256	0.9253 (2)	0.07830 (19)	0.30257 (18)	0.0189 (7)	
N261	0.73700 (17)	0.09295 (16)	0.14540 (14)	0.0188 (6)	
C262	0.6732 (2)	0.1446 (2)	0.12181 (19)	0.0255 (8)	
H262	0.6195	0.1223	0.0805	0.031*	
C263	0.6803 (2)	0.2279 (2)	0.15349 (19)	0.0279 (8)	
H263	0.6318	0.2613	0.1363	0.033*	
C264	0.7607 (2)	0.2612 (2)	0.21132 (19)	0.0242 (8)	
H264	0.7685	0.3185	0.2345	0.029*	
C265	0.8291 (2)	0.21070 (19)	0.23486 (17)	0.0200 (7)	
H265	0.8856	0.2328	0.2732	0.024*	
C266	0.8138 (2)	0.1269 (2)	0.20160 (17)	0.0175 (7)	

### Atomic displacement parameters (Å^2^)

**Table:**

	*U*^11^	*U*^22^	*U*^33^	*U*^12^	*U*^13^	*U*^23^
La1	0.01984 (11)	0.01524 (11)	0.01523 (11)	0.00090 (8)	0.00573 (9)	0.00149 (8)
O11	0.0287 (13)	0.0140 (12)	0.0118 (12)	−0.0005 (9)	0.0047 (10)	0.0019 (10)
C11	0.0182 (17)	0.0146 (18)	0.0135 (18)	−0.0008 (13)	0.0053 (14)	−0.0005 (14)
C12	0.0186 (17)	0.0147 (17)	0.021 (2)	−0.0002 (13)	0.0051 (15)	0.0013 (15)
C13	0.0160 (17)	0.025 (2)	0.030 (2)	0.0045 (14)	0.0088 (16)	0.0090 (17)
C14	0.0215 (17)	0.0109 (17)	0.029 (2)	0.0007 (13)	0.0093 (16)	0.0069 (15)
C15	0.0221 (18)	0.0173 (18)	0.026 (2)	0.0030 (14)	0.0123 (16)	0.0027 (15)
C16	0.0193 (17)	0.0178 (18)	0.0192 (19)	0.0009 (13)	0.0058 (15)	0.0034 (15)
N12	0.0276 (16)	0.0204 (17)	0.0254 (18)	−0.0024 (13)	0.0097 (14)	0.0065 (14)
O121	0.0415 (15)	0.0174 (13)	0.0232 (14)	0.0038 (11)	0.0181 (12)	0.0048 (11)
O122	0.0666 (19)	0.0282 (15)	0.0176 (15)	0.0025 (13)	0.0105 (14)	0.0083 (12)
N14	0.0156 (16)	0.0208 (17)	0.050 (2)	−0.0010 (13)	0.0167 (16)	−0.0034 (17)
O141	0.0266 (14)	0.0346 (16)	0.059 (2)	0.0072 (11)	0.0092 (14)	0.0413 (15)
O142	0.0330 (14)	0.0181 (14)	0.0419 (17)	0.0038 (11)	0.0164 (13)	0.0072 (12)
N16	0.0319 (17)	0.0166 (16)	0.0159 (16)	−0.0054 (13)	0.0049 (14)	−0.0033 (13)
O161	0.0430 (16)	0.0284 (15)	0.0223 (15)	0.0105 (12)	0.0052 (13)	0.0056 (12)
O162	0.0443 (16)	0.0328 (15)	0.0259 (15)	0.0027 (12)	0.0186 (13)	−0.0015 (12)
O21	0.0258 (13)	0.0267 (14)	0.0153 (13)	−0.0021 (10)	0.0111 (11)	−0.0025 (11)
C21	0.033 (2)	0.0173 (18)	0.026 (2)	−0.0058 (15)	0.0192 (18)	0.0026 (16)
C22	0.031 (2)	0.0229 (19)	0.0126 (19)	−0.0035 (15)	0.0101 (16)	0.0009 (15)
C23	0.045 (2)	0.0204 (19)	0.020 (2)	−0.0112 (17)	0.0147 (18)	−0.0022 (16)
C24	0.046 (2)	0.024 (2)	0.025 (2)	−0.0113 (17)	0.027 (2)	−0.0074 (16)
C25	0.040 (2)	0.018 (2)	0.044 (3)	−0.0038 (16)	0.029 (2)	−0.0016 (18)
C26	0.0218 (18)	0.0221 (19)	0.030 (2)	−0.0048 (15)	0.0130 (17)	0.0002 (16)
N22	0.0291 (18)	0.0249 (17)	0.0211 (19)	0.0020 (13)	0.0074 (15)	0.0036 (14)
O221	0.0267 (13)	0.0288 (14)	0.0185 (14)	0.0031 (10)	0.0056 (11)	0.0049 (11)
O222	0.0450 (17)	0.0543 (19)	0.0138 (15)	0.0095 (14)	−0.0029 (13)	0.0099 (13)
N24	0.075 (3)	0.0246 (19)	0.038 (2)	−0.0117 (19)	0.037 (2)	−0.0106 (17)
O241	0.079 (2)	0.063 (2)	0.035 (2)	−0.0212 (18)	0.0286 (19)	−0.0181 (17)
O242	0.083 (2)	0.049 (2)	0.063 (2)	0.0098 (17)	0.053 (2)	−0.0055 (17)
N26	0.0261 (18)	0.039 (2)	0.037 (2)	−0.0019 (16)	0.0168 (16)	0.0081 (18)
O261	0.0342 (15)	0.0360 (17)	0.0351 (17)	−0.0069 (12)	0.0152 (13)	−0.0028 (14)
O262	0.0366 (16)	0.0430 (18)	0.059 (2)	0.0121 (14)	0.0115 (15)	0.0115 (16)
O31	0.0229 (13)	0.0181 (13)	0.0240 (14)	0.0019 (10)	0.0019 (11)	0.0053 (11)
C31	0.027 (2)	0.0204 (19)	0.0133 (18)	0.0027 (15)	0.0064 (16)	0.0040 (15)
C32	0.0235 (18)	0.0128 (17)	0.0120 (18)	0.0038 (13)	0.0051 (15)	−0.0001 (14)
C33	0.0250 (19)	0.0184 (18)	0.0185 (19)	−0.0001 (14)	0.0062 (16)	0.0039 (15)
C34	0.0224 (19)	0.025 (2)	0.024 (2)	0.0021 (15)	0.0079 (16)	0.0043 (16)
C35	0.027 (2)	0.025 (2)	0.029 (2)	0.0093 (15)	0.0107 (17)	0.0106 (17)
C36	0.037 (2)	0.0097 (17)	0.028 (2)	0.0013 (15)	0.0110 (18)	0.0065 (15)
N32	0.0300 (17)	0.0149 (16)	0.0235 (17)	0.0049 (13)	0.0133 (15)	0.0039 (13)
O321	0.0219 (13)	0.0217 (13)	0.0265 (15)	0.0044 (10)	0.0044 (12)	0.0057 (11)
O322	0.0294 (13)	0.0155 (13)	0.0348 (16)	−0.0002 (10)	0.0107 (12)	0.0009 (11)
N34	0.0213 (16)	0.0269 (18)	0.0275 (18)	0.0019 (14)	0.0058 (14)	0.0114 (15)
O341	0.0224 (13)	0.0229 (14)	0.0389 (16)	−0.0030 (10)	0.0045 (12)	0.0094 (12)
O342	0.0241 (14)	0.0340 (16)	0.0488 (18)	0.0085 (12)	0.0119 (13)	0.0168 (14)
N36	0.0272 (18)	0.0194 (17)	0.048 (2)	0.0000 (14)	0.0021 (17)	0.0027 (16)
O361	0.022 (2)	0.0260 (18)	0.067 (6)	0.003 (2)	0.022 (3)	−0.006 (5)
O362	0.042 (4)	0.019 (3)	0.082 (6)	0.005 (2)	0.018 (4)	0.015 (3)
O363	0.022 (2)	0.0260 (18)	0.067 (6)	0.003 (2)	0.022 (3)	−0.006 (5)
O364	0.019 (4)	0.012 (3)	0.059 (8)	0.004 (3)	0.001 (4)	0.009 (4)
O011	0.0241 (13)	0.0253 (13)	0.0208 (14)	−0.0024 (10)	0.0121 (11)	−0.0032 (11)
C011	0.0196 (18)	0.026 (2)	0.026 (2)	−0.0030 (15)	0.0057 (16)	−0.0008 (17)
C012	0.061 (3)	0.044 (3)	0.047 (3)	−0.018 (2)	0.012 (2)	0.000 (2)
O021	0.0198 (12)	0.0189 (12)	0.0233 (14)	0.0015 (9)	0.0072 (11)	0.0058 (10)
C021	0.029 (2)	0.0134 (18)	0.028 (2)	−0.0052 (14)	0.0120 (17)	0.0018 (15)
C022	0.034 (2)	0.029 (2)	0.041 (3)	−0.0041 (17)	0.0141 (19)	0.0195 (19)
O031	0.0402 (15)	0.0219 (13)	0.0164 (13)	0.0101 (11)	0.0107 (12)	0.0039 (11)
C031	0.021 (3)	0.032 (3)	0.016 (3)	0.007 (2)	0.006 (2)	0.000 (3)
C032	0.027 (3)	0.032 (4)	0.018 (4)	0.001 (3)	−0.004 (3)	0.005 (3)
O033	0.0402 (15)	0.0219 (13)	0.0164 (13)	0.0101 (11)	0.0107 (12)	0.0039 (11)
C033	0.022 (6)	0.030 (6)	0.020 (6)	0.020 (5)	0.011 (5)	0.006 (5)
C034	0.020 (6)	0.069 (9)	0.015 (6)	−0.006 (6)	−0.005 (5)	0.006 (6)
N100	0.0185 (14)	0.0234 (16)	0.0115 (15)	−0.0043 (11)	0.0037 (12)	0.0028 (12)
N111	0.0232 (15)	0.0219 (16)	0.0180 (16)	0.0032 (12)	0.0089 (13)	0.0056 (13)
C112	0.0284 (19)	0.0150 (18)	0.034 (2)	0.0044 (14)	0.0125 (18)	0.0099 (16)
C113	0.0239 (19)	0.034 (2)	0.024 (2)	0.0075 (15)	0.0136 (16)	0.0175 (17)
C114	0.030 (2)	0.030 (2)	0.018 (2)	0.0013 (16)	0.0102 (16)	0.0059 (16)
C115	0.030 (2)	0.0195 (19)	0.023 (2)	0.0024 (15)	0.0090 (17)	0.0034 (16)
C116	0.0206 (17)	0.0169 (18)	0.0161 (18)	0.0019 (13)	0.0069 (15)	0.0044 (14)
N121	0.0236 (15)	0.0234 (16)	0.0195 (17)	0.0019 (12)	0.0081 (13)	0.0058 (13)
C122	0.030 (2)	0.029 (2)	0.017 (2)	0.0036 (16)	0.0106 (17)	0.0074 (16)
C123	0.029 (2)	0.034 (2)	0.019 (2)	−0.0006 (16)	0.0022 (17)	0.0055 (17)
C124	0.0208 (18)	0.025 (2)	0.032 (2)	0.0013 (15)	0.0029 (17)	0.0080 (17)
C125	0.0225 (18)	0.0203 (19)	0.024 (2)	−0.0001 (14)	0.0074 (16)	0.0058 (16)
C126	0.0266 (18)	0.0143 (17)	0.0156 (19)	0.0048 (14)	0.0088 (15)	0.0046 (14)
N131	0.0260 (16)	0.0170 (15)	0.0170 (16)	−0.0005 (12)	0.0080 (13)	−0.0002 (12)
C132	0.038 (2)	0.0204 (19)	0.018 (2)	0.0005 (16)	0.0139 (17)	−0.0024 (15)
C133	0.035 (2)	0.0207 (19)	0.0158 (19)	−0.0013 (15)	0.0100 (17)	0.0013 (15)
C134	0.041 (2)	0.0196 (19)	0.024 (2)	0.0007 (16)	0.0181 (18)	0.0082 (16)
C135	0.0276 (19)	0.0223 (19)	0.024 (2)	0.0068 (15)	0.0142 (16)	0.0091 (16)
C136	0.0244 (18)	0.0198 (18)	0.0119 (18)	0.0028 (14)	0.0101 (15)	0.0036 (14)
La2	0.01786 (10)	0.01719 (11)	0.01533 (11)	0.00167 (8)	0.00620 (9)	0.00436 (8)
O41	0.0261 (13)	0.0195 (13)	0.0140 (13)	0.0030 (10)	0.0061 (10)	0.0069 (10)
C41	0.0237 (19)	0.027 (2)	0.0170 (19)	0.0020 (15)	0.0061 (16)	0.0117 (16)
C42	0.0155 (16)	0.0200 (18)	0.0132 (18)	0.0021 (13)	0.0010 (14)	0.0057 (14)
C43	0.0212 (18)	0.0185 (18)	0.021 (2)	0.0008 (14)	0.0084 (15)	0.0004 (15)
C44	0.0115 (16)	0.0227 (19)	0.026 (2)	0.0031 (13)	0.0033 (15)	0.0081 (16)
C45	0.0245 (18)	0.0219 (19)	0.024 (2)	0.0028 (14)	0.0113 (16)	0.0119 (16)
C46	0.0181 (17)	0.0219 (19)	0.0117 (18)	0.0032 (14)	0.0048 (14)	0.0037 (14)
N42	0.0223 (15)	0.0203 (16)	0.0158 (16)	0.0039 (12)	0.0028 (13)	0.0050 (13)
O421	0.0303 (13)	0.0171 (13)	0.0244 (14)	0.0047 (10)	0.0142 (11)	0.0059 (11)
O422	0.0536 (17)	0.0242 (14)	0.0150 (14)	0.0003 (12)	0.0048 (13)	0.0034 (12)
N44	0.0181 (15)	0.0219 (17)	0.0276 (19)	0.0099 (12)	0.0111 (14)	0.0195 (15)
O441	0.0281 (14)	0.0190 (14)	0.052 (2)	0.0045 (11)	0.0117 (14)	0.0090 (14)
O442	0.0356 (15)	0.0219 (14)	0.0414 (17)	0.0079 (11)	0.0186 (14)	0.0142 (13)
N46	0.0283 (17)	0.0257 (17)	0.0257 (18)	0.0071 (14)	0.0062 (15)	0.0155 (15)
O461	0.0443 (16)	0.0310 (15)	0.0212 (15)	−0.0067 (12)	0.0075 (13)	0.0053 (12)
O462	0.0447 (16)	0.0469 (17)	0.0263 (16)	0.0057 (13)	0.0215 (14)	0.0098 (13)
O51	0.0227 (13)	0.0273 (14)	0.0172 (13)	0.0091 (10)	0.0092 (11)	0.0074 (11)
C51	0.0253 (18)	0.0133 (17)	0.023 (2)	0.0094 (14)	0.0154 (16)	0.0055 (15)
C52	0.0233 (19)	0.025 (2)	0.019 (2)	0.0030 (15)	0.0097 (16)	0.0064 (16)
C53	0.036 (2)	0.025 (2)	0.0134 (19)	0.0114 (16)	0.0105 (16)	0.0017 (15)
C54	0.042 (2)	0.028 (2)	0.020 (2)	0.0139 (17)	0.0205 (18)	0.0130 (16)
C55	0.034 (2)	0.029 (2)	0.032 (2)	0.0123 (16)	0.0259 (19)	0.0155 (17)
C56	0.0202 (18)	0.0233 (19)	0.030 (2)	0.0120 (14)	0.0131 (17)	0.0092 (16)
N52	0.0325 (18)	0.0217 (17)	0.0200 (18)	0.0098 (13)	0.0082 (15)	0.0054 (14)
O521	0.0253 (13)	0.0298 (14)	0.0162 (14)	0.0016 (10)	0.0101 (11)	0.0030 (11)
O522	0.0377 (15)	0.0375 (16)	0.0178 (15)	−0.0060 (12)	0.0000 (12)	−0.0020 (12)
N54	0.059 (2)	0.038 (2)	0.033 (2)	0.0226 (18)	0.033 (2)	0.0180 (17)
O541	0.072 (2)	0.072 (2)	0.0190 (17)	0.0246 (17)	0.0168 (16)	0.0239 (16)
O542	0.067 (2)	0.060 (2)	0.048 (2)	0.0144 (16)	0.0424 (17)	0.0273 (16)
N56	0.0198 (16)	0.042 (2)	0.0283 (19)	0.0070 (14)	0.0143 (15)	0.0080 (16)
O561	0.0278 (14)	0.0454 (17)	0.0285 (16)	0.0096 (12)	0.0090 (12)	0.0137 (14)
O562	0.0331 (15)	0.0415 (18)	0.056 (2)	−0.0073 (13)	0.0128 (14)	0.0125 (15)
O61	0.0164 (12)	0.0200 (13)	0.0191 (13)	−0.0003 (9)	0.0015 (10)	0.0020 (10)
C61	0.029 (2)	0.0175 (18)	0.0067 (17)	0.0013 (14)	0.0067 (15)	0.0016 (14)
C62	0.0248 (19)	0.0228 (19)	0.0176 (19)	−0.0008 (15)	0.0112 (16)	0.0053 (15)
C63	0.0270 (19)	0.0178 (18)	0.0156 (18)	0.0046 (14)	0.0095 (15)	0.0045 (14)
C64	0.0130 (16)	0.028 (2)	0.0081 (17)	0.0004 (14)	0.0019 (13)	0.0005 (14)
C65	0.0160 (17)	0.0231 (19)	0.0177 (19)	−0.0020 (14)	0.0074 (15)	0.0020 (15)
C66	0.0202 (18)	0.0207 (19)	0.0193 (19)	−0.0003 (14)	0.0034 (15)	0.0047 (15)
N62	0.0206 (16)	0.0155 (15)	0.0204 (16)	−0.0005 (12)	0.0061 (13)	0.0037 (13)
O621	0.0191 (13)	0.0212 (13)	0.0238 (14)	−0.0021 (10)	0.0026 (11)	0.0049 (11)
O622	0.0285 (14)	0.0266 (14)	0.0383 (16)	−0.0003 (11)	0.0035 (12)	0.0221 (13)
N64	0.0223 (16)	0.0223 (17)	0.0237 (18)	0.0031 (13)	0.0092 (14)	0.0048 (14)
O641	0.0236 (13)	0.0247 (14)	0.0284 (15)	0.0079 (10)	0.0060 (11)	0.0070 (12)
O642	0.0251 (13)	0.0275 (14)	0.0419 (17)	−0.0035 (11)	0.0120 (12)	0.0091 (12)
N66	0.0282 (17)	0.0233 (17)	0.036 (2)	0.0043 (14)	0.0143 (16)	0.0140 (15)
O661	0.0201 (13)	0.0354 (15)	0.0474 (18)	0.0043 (11)	0.0024 (13)	0.0239 (14)
O662	0.0396 (16)	0.0194 (14)	0.0534 (19)	−0.0042 (12)	0.0088 (14)	0.0108 (13)
O041	0.0208 (12)	0.0295 (13)	0.0157 (13)	0.0053 (10)	0.0078 (10)	0.0096 (11)
C041	0.0136 (17)	0.034 (2)	0.025 (2)	−0.0005 (15)	0.0003 (15)	0.0138 (17)
C042	0.052 (3)	0.046 (3)	0.047 (3)	0.031 (2)	0.012 (2)	0.018 (2)
O051	0.0216 (12)	0.0121 (11)	0.0206 (13)	0.0007 (9)	0.0084 (10)	−0.0027 (10)
C051	0.0234 (18)	0.0171 (19)	0.024 (2)	0.0079 (14)	0.0082 (16)	0.0048 (15)
C052	0.035 (2)	0.0184 (19)	0.027 (2)	0.0038 (15)	0.0124 (18)	−0.0104 (16)
O061	0.0280 (13)	0.0166 (12)	0.0167 (13)	−0.0024 (9)	0.0101 (11)	0.0010 (10)
C061	0.031 (2)	0.025 (2)	0.0096 (18)	−0.0022 (15)	0.0097 (16)	−0.0005 (15)
C062	0.027 (2)	0.030 (2)	0.022 (2)	0.0034 (16)	0.0104 (17)	0.0019 (17)
N200	0.0221 (15)	0.0206 (15)	0.0164 (16)	0.0046 (12)	0.0094 (13)	0.0045 (12)
N241	0.0189 (15)	0.0159 (15)	0.0224 (17)	0.0032 (11)	0.0097 (13)	0.0041 (13)
C242	0.0175 (17)	0.0177 (18)	0.029 (2)	0.0029 (14)	0.0037 (16)	0.0027 (16)
C243	0.0216 (18)	0.023 (2)	0.021 (2)	−0.0001 (14)	0.0065 (16)	−0.0075 (16)
C244	0.0273 (19)	0.029 (2)	0.0150 (19)	−0.0016 (15)	0.0105 (16)	0.0055 (16)
C245	0.0270 (18)	0.0154 (17)	0.0163 (19)	−0.0013 (14)	0.0087 (15)	0.0008 (14)
C246	0.0198 (17)	0.0177 (18)	0.0171 (19)	0.0016 (13)	0.0050 (15)	−0.0002 (15)
N251	0.0269 (16)	0.0219 (16)	0.0163 (16)	0.0029 (12)	0.0106 (13)	0.0012 (13)
C252	0.034 (2)	0.0238 (19)	0.0110 (18)	0.0002 (15)	0.0107 (16)	−0.0033 (15)
C253	0.037 (2)	0.027 (2)	0.0125 (19)	0.0038 (16)	−0.0023 (17)	0.0021 (16)
C254	0.0242 (19)	0.026 (2)	0.025 (2)	0.0025 (15)	0.0024 (17)	−0.0005 (17)
C255	0.0159 (17)	0.030 (2)	0.021 (2)	0.0053 (14)	0.0027 (15)	0.0079 (16)
C256	0.0256 (18)	0.0105 (17)	0.0165 (19)	0.0020 (13)	0.0052 (15)	−0.0008 (14)
N261	0.0222 (15)	0.0160 (15)	0.0147 (15)	0.0007 (11)	0.0035 (13)	0.0024 (12)
C262	0.0235 (19)	0.027 (2)	0.020 (2)	−0.0004 (15)	0.0011 (16)	0.0077 (16)
C263	0.029 (2)	0.031 (2)	0.028 (2)	0.0119 (16)	0.0104 (18)	0.0156 (18)
C264	0.0279 (19)	0.0153 (18)	0.028 (2)	0.0024 (14)	0.0100 (17)	0.0021 (16)
C265	0.0254 (18)	0.0165 (18)	0.0119 (18)	−0.0001 (14)	0.0026 (15)	−0.0018 (14)
C266	0.0239 (18)	0.0203 (18)	0.0129 (18)	0.0065 (14)	0.0083 (15)	0.0105 (14)

### Geometric parameters (Å, °)

**Table:**

La1—O11	2.418 (2)	C134—C135	1.379 (4)
La1—O31	2.422 (2)	C134—H134	0.9500
La1—O21	2.423 (2)	C135—C136	1.382 (4)
La1—O021	2.501 (2)	C135—H135	0.9500
La1—O011	2.507 (2)	La2—O41	2.404 (2)
La1—O031	2.508 (2)	La2—O61	2.415 (2)
La1—O121	2.623 (2)	La2—O51	2.423 (2)
La1—O321	2.648 (2)	La2—O041	2.506 (2)
La1—O221	2.758 (2)	La2—O051	2.515 (2)
O11—C11	1.273 (3)	La2—O061	2.516 (2)
C11—C12	1.431 (4)	La2—O421	2.624 (2)
C11—C16	1.443 (4)	La2—O621	2.702 (2)
C12—C13	1.373 (4)	La2—O521	2.759 (2)
C12—N12	1.454 (4)	O41—C41	1.272 (4)
C13—C14	1.369 (4)	C41—C42	1.432 (4)
C13—H13	0.9500	C41—C46	1.435 (4)
C14—C15	1.388 (5)	C42—C43	1.381 (4)
C14—N14	1.471 (4)	C42—N42	1.461 (4)
C15—C16	1.357 (4)	C43—C44	1.378 (4)
C15—H15	0.9500	C43—H43	0.9500
C16—N16	1.476 (4)	C44—C45	1.397 (5)
N12—O122	1.228 (4)	C44—N44	1.504 (4)
N12—O121	1.240 (3)	C45—C46	1.361 (4)
N14—O141	1.183 (4)	C45—H45	0.9500
N14—O142	1.245 (4)	C46—N46	1.474 (4)
N16—O161	1.226 (3)	N42—O422	1.220 (3)
N16—O162	1.234 (3)	N42—O421	1.245 (3)
O21—C21	1.265 (4)	N44—O441	1.175 (3)
C21—C22	1.431 (5)	N44—O442	1.223 (3)
C21—C26	1.435 (5)	N46—O461	1.223 (3)
C22—C23	1.387 (4)	N46—O462	1.226 (3)
C22—N22	1.465 (4)	O51—C51	1.270 (4)
C23—C24	1.378 (5)	C51—C52	1.430 (5)
C23—H23	0.9500	C51—C56	1.435 (4)
C24—C25	1.384 (5)	C52—C53	1.387 (4)
C24—N24	1.467 (4)	C52—N52	1.447 (4)
C25—C26	1.369 (5)	C53—C54	1.375 (5)
C25—H25	0.9500	C53—H53	0.9500
C26—N26	1.474 (5)	C54—C55	1.381 (5)
N22—O222	1.220 (4)	C54—N54	1.461 (4)
N22—O221	1.236 (3)	C55—C56	1.377 (4)
N24—O241	1.225 (4)	C55—H55	0.9500
N24—O242	1.236 (4)	C56—N56	1.461 (4)
N26—O262	1.226 (4)	N52—O522	1.234 (3)
N26—O261	1.229 (4)	N52—O521	1.238 (3)
O31—C31	1.272 (4)	N54—O541	1.218 (4)
C31—C36	1.439 (5)	N54—O542	1.240 (4)
C31—C32	1.442 (4)	N56—O561	1.230 (4)
C32—C33	1.377 (4)	N56—O562	1.231 (4)
C32—N32	1.463 (4)	O61—C61	1.263 (4)
C33—C34	1.360 (4)	C61—C66	1.444 (4)
C33—H33	0.9500	C61—C62	1.450 (5)
C34—C35	1.388 (4)	C62—C63	1.386 (4)
C34—N34	1.464 (4)	C62—N62	1.456 (4)
C35—C36	1.369 (5)	C63—C64	1.395 (4)
C35—H35	0.9500	C63—H63	0.9500
C36—N36	1.468 (4)	C64—C65	1.384 (4)
N32—O322	1.232 (3)	C64—N64	1.456 (4)
N32—O321	1.242 (3)	C65—C66	1.361 (4)
N34—O341	1.225 (3)	C65—H65	0.9500
N34—O342	1.237 (3)	C66—N66	1.473 (4)
N36—O363	1.214 (12)	N62—O622	1.237 (3)
N36—O361	1.227 (9)	N62—O621	1.239 (3)
N36—O362	1.244 (7)	N64—O641	1.223 (3)
N36—O364	1.282 (9)	N64—O642	1.237 (3)
O011—C011	1.453 (4)	N66—O662	1.229 (3)
O011—H011	0.8400	N66—O661	1.231 (3)
C011—C012	1.484 (5)	O041—C041	1.445 (4)
C011—H01A	0.9900	O041—H041	0.8400
C011—H01B	0.9900	C041—C042	1.512 (5)
C012—H01C	0.9800	C041—H04A	0.9900
C012—H01D	0.9800	C041—H04B	0.9900
C012—H01E	0.9800	C042—H04C	0.9800
O021—C021	1.446 (3)	C042—H04D	0.9800
O021—H021	0.8400	C042—H04E	0.9800
C021—C022	1.501 (5)	O051—C051	1.441 (4)
C021—H02A	0.9900	O051—H051	0.8402
C021—H02B	0.9900	C051—C052	1.499 (4)
C022—H02C	0.9800	C051—H05A	0.9900
C022—H02D	0.9800	C051—H05B	0.9900
C022—H02E	0.9800	C052—H05C	0.9800
O031—C031	1.476 (6)	C052—H05D	0.9800
O031—H031	0.8396	C052—H05E	0.9800
O031—H033	0.8393	O061—C061	1.439 (4)
C031—C032	1.488 (7)	O061—H061	0.8401
C031—H03A	0.9900	C061—C062	1.514 (4)
C031—H03B	0.9900	C061—H06A	0.9900
C032—H03C	0.9800	C061—H06B	0.9900
C032—H03D	0.9800	C062—H06C	0.9800
C032—H03E	0.9800	C062—H06D	0.9800
C033—C034	1.489 (12)	C062—H06E	0.9800
C033—H03F	0.9900	N200—C246	1.421 (4)
C033—H03G	0.9900	N200—C256	1.424 (4)
C034—H03H	0.9800	N200—C266	1.426 (4)
C034—H03I	0.9800	N241—C246	1.336 (4)
C034—H03J	0.9800	N241—C242	1.352 (4)
N100—C126	1.415 (4)	C242—C243	1.379 (5)
N100—C136	1.426 (4)	C242—H242	0.9500
N100—C116	1.428 (4)	C243—C244	1.377 (5)
N111—C116	1.334 (4)	C243—H243	0.9500
N111—C112	1.349 (4)	C244—C245	1.382 (4)
C112—C113	1.378 (5)	C244—H244	0.9500
C112—H112	0.9500	C245—C246	1.379 (4)
C113—C114	1.376 (5)	C245—H245	0.9500
C113—H113	0.9500	N251—C256	1.325 (4)
C114—C115	1.383 (4)	N251—C252	1.343 (4)
C114—H114	0.9500	C252—C253	1.366 (5)
C115—C116	1.382 (4)	C252—H252	0.9500
C115—H115	0.9500	C253—C254	1.390 (5)
N121—C126	1.340 (4)	C253—H253	0.9500
N121—C122	1.340 (4)	C254—C255	1.379 (5)
C122—C123	1.368 (5)	C254—H254	0.9500
C122—H122	0.9500	C255—C256	1.400 (4)
C123—C124	1.393 (5)	C255—H255	0.9500
C123—H123	0.9500	N261—C266	1.340 (4)
C124—C125	1.379 (5)	N261—C262	1.341 (4)
C124—H124	0.9500	C262—C263	1.377 (5)
C125—C126	1.392 (4)	C262—H262	0.9500
C125—H125	0.9500	C263—C264	1.386 (5)
N131—C136	1.336 (4)	C263—H263	0.9500
N131—C132	1.356 (4)	C264—C265	1.373 (4)
C132—C133	1.369 (5)	C264—H264	0.9500
C132—H132	0.9500	C265—C266	1.384 (4)
C133—C134	1.379 (5)	C265—H265	0.9500
C133—H133	0.9500		
			
O11···O011	3.217 (3)	O41···O041	3.115 (4)
O21···O021	3.341 (3)	O51···O051	3.397 (4)
O31···O031	3.400 (4)	O61···O061	3.483 (4)
			
O11—La1—O31	83.56 (7)	C133—C134—H134	120.0
O11—La1—O21	77.24 (7)	C134—C135—C136	117.7 (3)
O31—La1—O21	78.13 (8)	C134—C135—H135	121.2
O11—La1—O021	135.75 (7)	C136—C135—H135	121.2
O31—La1—O021	132.33 (7)	N131—C136—C135	123.7 (3)
O21—La1—O021	85.43 (7)	N131—C136—N100	115.6 (3)
O11—La1—O011	81.53 (7)	C135—C136—N100	120.6 (3)
O31—La1—O011	140.19 (7)	O41—La2—O61	84.07 (7)
O21—La1—O011	132.99 (7)	O41—La2—O51	78.16 (7)
O021—La1—O011	81.35 (7)	O61—La2—O51	79.12 (7)
O11—La1—O031	132.41 (7)	O41—La2—O041	78.73 (7)
O31—La1—O031	87.21 (8)	O61—La2—O041	139.58 (7)
O21—La1—O031	145.42 (7)	O51—La2—O041	131.15 (7)
O021—La1—O031	81.64 (7)	O41—La2—O051	136.73 (7)
O011—La1—O031	76.40 (7)	O61—La2—O051	132.83 (7)
O11—La1—O121	64.93 (7)	O51—La2—O051	86.93 (7)
O31—La1—O121	67.28 (7)	O041—La2—O051	81.41 (7)
O21—La1—O121	130.43 (7)	O41—La2—O061	132.65 (7)
O021—La1—O121	144.13 (7)	O61—La2—O061	89.85 (7)
O011—La1—O121	72.96 (7)	O51—La2—O061	146.34 (7)
O031—La1—O121	68.43 (7)	O041—La2—O061	76.13 (7)
O11—La1—O321	139.95 (7)	O051—La2—O061	77.46 (7)
O31—La1—O321	63.77 (7)	O41—La2—O421	65.29 (7)
O21—La1—O321	74.00 (7)	O61—La2—O421	66.78 (7)
O021—La1—O321	68.73 (7)	O51—La2—O421	131.44 (7)
O011—La1—O321	138.46 (7)	O041—La2—O421	72.81 (7)
O031—La1—O321	71.43 (7)	O051—La2—O421	141.63 (7)
O121—La1—O321	116.62 (7)	O061—La2—O421	69.19 (7)
O11—La1—O221	70.14 (7)	O41—La2—O621	139.50 (7)
O31—La1—O221	136.22 (7)	O61—La2—O621	62.92 (7)
O21—La1—O221	62.61 (7)	O51—La2—O621	73.33 (7)
O021—La1—O221	65.73 (7)	O041—La2—O621	141.71 (7)
O011—La1—O221	70.84 (7)	O051—La2—O621	69.92 (7)
O031—La1—O221	136.31 (7)	O061—La2—O621	73.33 (7)
O121—La1—O221	125.17 (7)	O421—La2—O621	115.75 (7)
O321—La1—O221	117.88 (7)	O41—La2—O521	71.61 (7)
C11—O11—La1	141.63 (19)	O61—La2—O521	137.79 (7)
O11—C11—C12	126.4 (3)	O51—La2—O521	62.72 (7)
O11—C11—C16	121.0 (3)	O041—La2—O521	69.31 (7)
C12—C11—C16	112.6 (3)	O051—La2—O521	65.47 (7)
C13—C12—C11	123.2 (3)	O061—La2—O521	131.97 (6)
C13—C12—N12	116.6 (3)	O421—La2—O521	126.73 (7)
C11—C12—N12	120.3 (3)	O621—La2—O521	117.28 (7)
C14—C13—C12	119.7 (3)	C41—O41—La2	142.7 (2)
C14—C13—H13	120.1	O41—C41—C42	125.7 (3)
C12—C13—H13	120.1	O41—C41—C46	120.9 (3)
C13—C14—C15	121.3 (3)	C42—C41—C46	113.4 (3)
C13—C14—N14	118.3 (3)	C43—C42—C41	123.1 (3)
C15—C14—N14	120.3 (3)	C43—C42—N42	115.6 (3)
C16—C15—C14	118.4 (3)	C41—C42—N42	121.3 (3)
C16—C15—H15	120.8	C44—C43—C42	119.0 (3)
C14—C15—H15	120.8	C44—C43—H43	120.5
C15—C16—C11	124.7 (3)	C42—C43—H43	120.5
C15—C16—N16	118.8 (3)	C43—C44—C45	121.7 (3)
C11—C16—N16	116.5 (3)	C43—C44—N44	118.2 (3)
O122—N12—O121	121.9 (3)	C45—C44—N44	120.1 (3)
O122—N12—C12	118.9 (3)	C46—C45—C44	118.0 (3)
O121—N12—C12	119.1 (3)	C46—C45—H45	121.0
N12—O121—La1	133.32 (19)	C44—C45—H45	121.0
O141—N14—O142	123.4 (3)	C45—C46—C41	124.6 (3)
O141—N14—C14	121.0 (3)	C45—C46—N46	118.9 (3)
O142—N14—C14	115.6 (3)	C41—C46—N46	116.4 (3)
O161—N16—O162	124.4 (3)	O422—N42—O421	121.8 (3)
O161—N16—C16	118.7 (3)	O422—N42—C42	119.2 (3)
O162—N16—C16	116.9 (3)	O421—N42—C42	118.9 (3)
C21—O21—La1	132.25 (19)	N42—O421—La2	135.20 (18)
O21—C21—C22	125.0 (3)	O441—N44—O442	128.1 (3)
O21—C21—C26	122.7 (3)	O441—N44—C44	117.6 (3)
C22—C21—C26	112.3 (3)	O442—N44—C44	114.2 (3)
C23—C22—C21	124.5 (3)	O461—N46—O462	125.2 (3)
C23—C22—N22	115.8 (3)	O461—N46—C46	117.6 (3)
C21—C22—N22	119.7 (3)	O462—N46—C46	117.2 (3)
C24—C23—C22	118.1 (3)	C51—O51—La2	133.24 (19)
C24—C23—H23	121.0	O51—C51—C52	125.6 (3)
C22—C23—H23	121.0	O51—C51—C56	121.5 (3)
C23—C24—C25	121.8 (3)	C52—C51—C56	112.9 (3)
C23—C24—N24	118.5 (4)	C53—C52—C51	123.9 (3)
C25—C24—N24	119.7 (4)	C53—C52—N52	116.7 (3)
C26—C25—C24	118.6 (3)	C51—C52—N52	119.4 (3)
C26—C25—H25	120.7	C54—C53—C52	118.8 (3)
C24—C25—H25	120.7	C54—C53—H53	120.6
C25—C26—C21	124.6 (3)	C52—C53—H53	120.6
C25—C26—N26	116.6 (3)	C53—C54—C55	121.4 (3)
C21—C26—N26	118.8 (3)	C53—C54—N54	118.8 (3)
O222—N22—O221	122.7 (3)	C55—C54—N54	119.7 (3)
O222—N22—C22	118.5 (3)	C56—C55—C54	119.0 (3)
O221—N22—C22	118.8 (3)	C56—C55—H55	120.5
N22—O221—La1	137.2 (2)	C54—C55—H55	120.5
O241—N24—O242	124.9 (4)	C55—C56—C51	123.8 (3)
O241—N24—C24	117.8 (4)	C55—C56—N56	117.8 (3)
O242—N24—C24	117.3 (4)	C51—C56—N56	118.2 (3)
O262—N26—O261	125.1 (3)	O522—N52—O521	122.1 (3)
O262—N26—C26	117.2 (3)	O522—N52—C52	118.0 (3)
O261—N26—C26	117.7 (3)	O521—N52—C52	119.9 (3)
C31—O31—La1	142.0 (2)	N52—O521—La2	136.9 (2)
O31—C31—C36	122.9 (3)	O541—N54—O542	124.1 (3)
O31—C31—C32	124.0 (3)	O541—N54—C54	118.5 (3)
C36—C31—C32	113.0 (3)	O542—N54—C54	117.3 (4)
C33—C32—C31	123.0 (3)	O561—N56—O562	123.6 (3)
C33—C32—N32	115.7 (3)	O561—N56—C56	118.7 (3)
C31—C32—N32	121.2 (3)	O562—N56—C56	117.6 (3)
C34—C33—C32	119.6 (3)	C61—O61—La2	141.8 (2)
C34—C33—H33	120.2	O61—C61—C66	123.7 (3)
C32—C33—H33	120.2	O61—C61—C62	124.1 (3)
C33—C34—C35	121.9 (3)	C66—C61—C62	112.0 (3)
C33—C34—N34	119.0 (3)	C63—C62—C61	124.2 (3)
C35—C34—N34	119.1 (3)	C63—C62—N62	115.4 (3)
C36—C35—C34	118.6 (3)	C61—C62—N62	120.4 (3)
C36—C35—H35	120.7	C62—C63—C64	118.2 (3)
C34—C35—H35	120.7	C62—C63—H63	120.9
C35—C36—C31	123.9 (3)	C64—C63—H63	120.9
C35—C36—N36	115.7 (3)	C65—C64—C63	121.6 (3)
C31—C36—N36	120.5 (3)	C65—C64—N64	119.4 (3)
O322—N32—O321	121.2 (3)	C63—C64—N64	119.0 (3)
O322—N32—C32	118.5 (3)	C66—C65—C64	119.1 (3)
O321—N32—C32	120.2 (3)	C66—C65—H65	120.4
N32—O321—La1	142.00 (19)	C64—C65—H65	120.4
O341—N34—O342	124.2 (3)	C65—C66—C61	124.9 (3)
O341—N34—C34	118.8 (3)	C65—C66—N66	116.8 (3)
O342—N34—C34	117.0 (3)	C61—C66—N66	118.3 (3)
O361—N36—O362	121.4 (12)	O622—N62—O621	121.7 (3)
O363—N36—O364	125.0 (16)	O622—N62—C62	118.1 (3)
O363—N36—C36	119.4 (17)	O621—N62—C62	120.3 (3)
O361—N36—C36	119.5 (12)	N62—O621—La2	140.69 (18)
O362—N36—C36	118.6 (4)	O641—N64—O642	123.7 (3)
O364—N36—C36	114.4 (5)	O641—N64—C64	119.0 (3)
C011—O011—La1	129.85 (18)	O642—N64—C64	117.3 (3)
C011—O011—H011	109.5	O662—N66—O661	123.3 (3)
La1—O011—H011	120.5	O662—N66—C66	117.0 (3)
O011—C011—C012	112.2 (3)	O661—N66—C66	119.6 (3)
O011—C011—H01A	109.2	C041—O041—La2	131.51 (17)
C012—C011—H01A	109.2	C041—O041—H041	109.5
O011—C011—H01B	109.2	La2—O041—H041	118.6
C012—C011—H01B	109.2	O041—C041—C042	112.2 (3)
H01A—C011—H01B	107.9	O041—C041—H04A	109.2
C011—C012—H01C	109.5	C042—C041—H04A	109.2
C011—C012—H01D	109.5	O041—C041—H04B	109.2
H01C—C012—H01D	109.5	C042—C041—H04B	109.2
C011—C012—H01E	109.5	H04A—C041—H04B	107.9
H01C—C012—H01E	109.5	C041—C042—H04C	109.5
H01D—C012—H01E	109.5	C041—C042—H04D	109.5
C021—O021—La1	127.51 (18)	H04C—C042—H04D	109.5
C021—O021—H021	109.5	C041—C042—H04E	109.5
La1—O021—H021	119.5	H04C—C042—H04E	109.5
O021—C021—C022	112.0 (3)	H04D—C042—H04E	109.5
O021—C021—H02A	109.2	C051—O051—La2	126.94 (17)
C022—C021—H02A	109.2	C051—O051—H051	109.5
O021—C021—H02B	109.2	La2—O051—H051	118.4
C022—C021—H02B	109.2	O051—C051—C052	111.7 (3)
H02A—C021—H02B	107.9	O051—C051—H05A	109.3
C021—C022—H02C	109.5	C052—C051—H05A	109.3
C021—C022—H02D	109.5	O051—C051—H05B	109.3
H02C—C022—H02D	109.5	C052—C051—H05B	109.3
C021—C022—H02E	109.5	H05A—C051—H05B	107.9
H02C—C022—H02E	109.5	C051—C052—H05C	109.5
H02D—C022—H02E	109.5	C051—C052—H05D	109.5
C031—O031—La1	138.2 (2)	H05C—C052—H05D	109.5
C031—O031—H031	109.4	C051—C052—H05E	109.5
La1—O031—H031	109.9	H05C—C052—H05E	109.5
C031—O031—H033	103.8	H05D—C052—H05E	109.5
La1—O031—H033	117.8	C061—O061—La2	135.52 (17)
O031—C031—C032	106.2 (4)	C061—O061—H061	109.5
O031—C031—H03A	110.5	La2—O061—H061	113.9
C032—C031—H03A	110.5	O061—C061—C062	110.5 (3)
O031—C031—H03B	110.5	O061—C061—H06A	109.6
C032—C031—H03B	110.5	C062—C061—H06A	109.6
H03A—C031—H03B	108.7	O061—C061—H06B	109.5
C031—C032—H03C	109.5	C062—C061—H06B	109.5
C031—C032—H03D	109.5	H06A—C061—H06B	108.1
H03C—C032—H03D	109.5	C061—C062—H06C	109.5
C031—C032—H03E	109.5	C061—C062—H06D	109.5
H03C—C032—H03E	109.5	H06C—C062—H06D	109.5
H03D—C032—H03E	109.5	C061—C062—H06E	109.5
C034—C033—H03F	110.2	H06C—C062—H06E	109.5
C034—C033—H03G	110.2	H06D—C062—H06E	109.5
H03F—C033—H03G	108.5	C246—N200—C256	120.8 (3)
C033—C034—H03H	109.5	C246—N200—C266	119.9 (3)
C033—C034—H03I	109.5	C256—N200—C266	118.8 (3)
H03H—C034—H03I	109.5	C246—N241—C242	117.3 (3)
C033—C034—H03J	109.5	N241—C242—C243	123.0 (3)
H03H—C034—H03J	109.5	N241—C242—H242	118.5
H03I—C034—H03J	109.5	C243—C242—H242	118.5
C126—N100—C136	119.9 (2)	C244—C243—C242	117.9 (3)
C126—N100—C116	120.2 (2)	C244—C243—H243	121.0
C136—N100—C116	119.8 (3)	C242—C243—H243	121.0
C116—N111—C112	116.8 (3)	C243—C244—C245	120.5 (3)
N111—C112—C113	123.4 (3)	C243—C244—H244	119.8
N111—C112—H112	118.3	C245—C244—H244	119.8
C113—C112—H112	118.3	C246—C245—C244	117.4 (3)
C114—C113—C112	118.6 (3)	C246—C245—H245	121.3
C114—C113—H113	120.7	C244—C245—H245	121.3
C112—C113—H113	120.7	N241—C246—C245	123.9 (3)
C113—C114—C115	119.2 (3)	N241—C246—N200	115.4 (3)
C113—C114—H114	120.4	C245—C246—N200	120.7 (3)
C115—C114—H114	120.4	C256—N251—C252	117.7 (3)
C114—C115—C116	118.3 (3)	N251—C252—C253	123.4 (3)
C114—C115—H115	120.9	N251—C252—H252	118.3
C116—C115—H115	120.9	C253—C252—H252	118.3
N111—C116—C115	123.7 (3)	C252—C253—C254	118.4 (3)
N111—C116—N100	115.1 (3)	C252—C253—H253	120.8
C115—C116—N100	121.2 (3)	C254—C253—H253	120.8
C126—N121—C122	117.8 (3)	C255—C254—C253	119.5 (3)
N121—C122—C123	123.6 (3)	C255—C254—H254	120.3
N121—C122—H122	118.2	C253—C254—H254	120.3
C123—C122—H122	118.2	C254—C255—C256	117.6 (3)
C122—C123—C124	118.5 (3)	C254—C255—H255	121.2
C122—C123—H123	120.8	C256—C255—H255	121.2
C124—C123—H123	120.8	N251—C256—C255	123.3 (3)
C125—C124—C123	118.9 (3)	N251—C256—N200	117.7 (3)
C125—C124—H124	120.5	C255—C256—N200	119.0 (3)
C123—C124—H124	120.5	C266—N261—C262	116.5 (3)
C124—C125—C126	118.7 (3)	N261—C262—C263	124.3 (3)
C124—C125—H125	120.6	N261—C262—H262	117.9
C126—C125—H125	120.6	C263—C262—H262	117.9
N121—C126—C125	122.4 (3)	C262—C263—C264	117.7 (3)
N121—C126—N100	116.5 (3)	C262—C263—H263	121.1
C125—C126—N100	121.0 (3)	C264—C263—H263	121.1
C136—N131—C132	117.0 (3)	C265—C264—C263	119.4 (3)
N131—C132—C133	123.2 (3)	C265—C264—H264	120.3
N131—C132—H132	118.4	C263—C264—H264	120.3
C133—C132—H132	118.4	C264—C265—C266	118.6 (3)
C132—C133—C134	118.3 (3)	C264—C265—H265	120.7
C132—C133—H133	120.8	C266—C265—H265	120.7
C134—C133—H133	120.8	N261—C266—C265	123.3 (3)
C135—C134—C133	120.1 (3)	N261—C266—N200	115.4 (3)
C135—C134—H134	120.0	C265—C266—N200	121.3 (3)
			
N111—C116—N100—C126	42.2 (4)	N241—C246—N200—C256	41.8 (4)
N121—C126—N100—C136	38.1 (4)	N251—C256—N200—C266	36.9 (4)
N131—C136—N100—C116	40.1 (4)	N261—C266—N200—C246	34.0 (5)

### Hydrogen-bond geometry (Å, °)

**Table:**

*D*—H···*A*	*D*—H	H···*A*	*D*···*A*	*D*—H···*A*
O011—H011···N111	0.839	1.902	2.740 (4)	175
O021—H021···N121	0.840	1.916	2.747 (3)	170
O031—H031···N131	0.840	1.895	2.693 (3)	158
O041—H041···N241^i^	0.839	1.881	2.721 (4)	179
O051—H051···N251^i^	0.841	1.934	2.769 (3)	172
O061—H061···N261^i^	0.840	1.854	2.692 (4)	175

Symmetry codes:  (i) *x*, *y*+1, *z*.

## References

[bb1] Altomare, A., Cascarano, G., Giacovazzo, C., Guagliardi, A., Burla, M. C., Polidori, G. & Camalli, M. (1994). *J. Appl. Cryst.* **27**, 435.

[bb2] Chan, E. J. (2006). Doctoral Thesis, School of Biomedical and Chemical Sciences, UWA, Australia.

[bb3] Hall, S. R., du Boulay, D. J. & Olthof-Hazekamp, R. (2001). *The Xtal3.7 System* The University of Western Australia, Australia.

[bb4] Harrowfield, J. (1996). *J. Chem. Soc. Dalton Trans.* pp. 3165–3171.

[bb5] Harrowfield, J. M., Skelton, B. W. & White, A. H. (1994). *Aust. J. Chem.* **47**, 359–364.

[bb6] Kepert, D. L. (1986). *Inorganic Stereochemistry.* Berlin: Springer-Verlag.

[bb7] Nagao, N., Egashira, K. & Mogi, D. (2004). *Bull. Chem. Soc. Jpn*, **77**, 1171–1172.

[bb8] Oxford Diffraction (2006). *CrysAlis CCD *and**CrysAlis RED** Oxford Diffraction Ltd, Abingdon, Oxfordshire, England.

[bb9] Sheldrick, G. M. (2008). *Acta Cryst.* A**64**, 112–122. 10.1107/S0108767307043930 18156677

